# Gastrointestinal Bezoars in Children: Clinical Characteristics, Contributing Factors, and Treatment Outcomes

**DOI:** 10.3390/children13070929

**Published:** 2026-07-15

**Authors:** Chia-Ying Lin, Mi-Chi Chen, En-Shuo Chang, Wan-Hsin Su, Pai-Jui Yeh, Ming-Wei Lai, Chien-Chang Chen, Hsun-Chin Chao

**Affiliations:** 1 Division of Pediatric Gastroenterology, Department of Pediatrics, Chang Gung Children’s Medical Center, Chang Gung Memorial Hospital, Linkou Branch, Taoyuan City 333423, Taiwan; 2 Graduate Institute of Clinical Medical Sciences, College of Medicine, Chang Gung University, Taoyuan City 333323, Taiwan; 3 Faculty Member, College of Medicine, Chang Gung University, Taoyuan City 333323, Taiwan

**Keywords:** bezoar, gastrointestinal, children

## Abstract

**Highlights:**

**What are the main findings?**

- Phytobezoars were the most frequent type in children, with the stomach identified as the most common location for bezoar formation in pediatric patients.
- A significant majority of patients had underlying medical conditions, including gastrointestinal anomalies and neuropsychiatric disorders.

**What are the implications of the main findings?**

- Clinical teams should anticipate a higher likelihood of surgery for patients with trichobezoars, bezoars located in the lower gastrointestinal tract, or those presenting with larger masses.

**Abstract:**

Background/Objectives: Bezoars are described as masses of undigested substances that can be found within the gastrointestinal tract. This study aimed to explore the types of bezoars observed in pediatric patients and examine the underlying factors contributing to the formation of these bezoars and treatment outcomes. Method: This retrospective study was conducted at a tertiary medical center in Northern Taiwan from January 2000 to June 2023. We enrolled pediatric patients diagnosed with bezoars in the gastrointestinal tract from the medical database of esophagogastroduodenoscopy, upper gastrointestinal series, lower gastrointestinal series, abdominal computed tomography, and surgical procedural codes. Demographic characteristics, presenting symptoms, imaging findings, and clinical course were reviewed. Results: A total of 25 patients, including 14 females (56%), with a median age of 12.0 years (interquartile range: 5.9–15.0 years), were enrolled in this study. Phytobezoars were observed in 17 patients, while trichobezoars were found in 5 patients, and 3 patients had lactobezoars. The stomach was identified as the most frequent site. Among these patients, gastrointestinal anomalies were found in 15 patients (60%), with neuropsychiatric disorders in 11 patients (44%). Surgical intervention was required for 14 patients (56%), 5 patients (20%) underwent endoscopic treatment, and 6 patients (24%) passed the bezoar spontaneously. Trichobezoars (100%, *p* = 0.040), as well as bezoars of larger size (*p* = 0.005) or those located in the lower gastrointestinal tract (57%, *p* = 0.033), were associated with a significantly higher likelihood of requiring surgical intervention. Conclusions: Phytobezoars are the predominant type of bezoar observed in children, with gastrointestinal anomalies and neuropsychiatric disorders being potential contributing factors. Patients with trichobezoars or bezoars located in the lower gastrointestinal tract, especially those of larger size, are more likely to require surgery for treatment.

## 1. Introduction

A bezoar is an undigested mass that accumulates in the gastrointestinal (GI) tract. Bezoars can be classified into five types: phytobezoar, composed of indigestible plant fibers; trichobezoar, composed of hair; lactobezoar, composed of milk protein; pharmacobezoar, composed of medications; and lithobezoar, composed of stone-like or calcified materials [1].

Bezoars are not commonly encountered, and their incidence varies, according to different studies. In addition, the prevalence of bezoars in pediatric populations remains unclear. While early studies by Kadian et al. (1978) and Ahn et al. (1987) reported a consistent incidence of 0.43% during esophagogastroduodenoscopy (EGD) screenings, more recent long-term data from Mihai et al. (2013) suggest a significantly lower rate of 0.068% [2,3,4]. Bezoars, which can be found anywhere in the GI tract, are most frequently located in the stomach [5,6]. However, they may also be found in the small intestine or even the large intestine. Yakan et al. identified 3.2% of cases (14 out of 432) with small bowel obstruction due to phytobezoars over 10 years [7]. Ghosheh et al. found bezoars as the fifth most common cause of acute small bowel obstruction (0.8% of 1061 cases) [8].

Phytobezoars have been identified as the most common type in previous studies [9], a trend also observed in children. In a retrospective study by Shah et al., which included 30 pediatric patients aged 2 to 18 years diagnosed with gastric bezoars, the majority of patients were found to have phytobezoars, with only one patient presenting with a trichobezoar [10]. Another retrospective study conducted by Dörterler et al., involving 16 pediatric patients who underwent endoscopy and/or surgical treatment for bezoars between 2011 and 2019, reported that phytobezoars were the predominant type (62.5%), followed by trichobezoars (12.5%) [6].

Several risk factors for bezoar formation have been proposed, including a high-fiber diet, undigested milk products, some pharmaceutical agents—especially bulk-forming hygroscopic laxatives or extended-release medicines coated with cellulose acetate to slow dissolution time—and impaired mastication [11]. Other underlying medical conditions, including GI tract anomalies and neuropsychiatric disorders such as intellectual disability and pica, have also been reported [6]. Among these psychological substrates, trichotillomania and trichophagia carry a unique and strong association with pediatric trichobezoars. Because hair resists gastric digestion and peristalsis, these entities typically accumulate over extended periods, leading to a characteristically delayed presentation of symptoms. Consequently, patients often remain asymptomatic until significant complications arise, such as chronic nutritional deficiencies, bowel obstruction, or palpable abdominal masses. While smaller aggregates may occasionally be managed with conservative or endoscopic approaches, large and impacted trichobezoars frequently mandate surgical intervention via laparotomy to prevent gastrointestinal obstruction or perforation [12].

A review of the literature reveals that previous studies have underemphasized optimal management strategies for pediatric bezoars. Additionally, clinical data regarding this condition remain scarce within Asian populations. To address these knowledge gaps, this retrospective single-center study aimed to characterize the diverse types of pediatric bezoars, identify their underlying medical conditions, and delineate the clinical features associated with the need for surgical intervention.

## 2. Materials and Methods

### 2.1. Study Design

**Figure 1:**
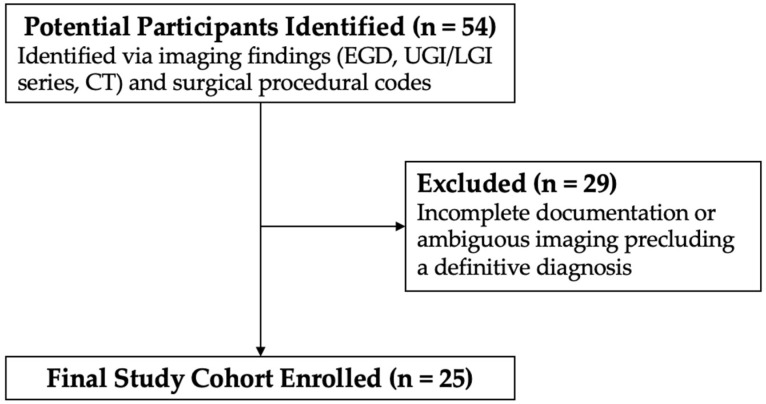
Flowchart of participant selection and enrollment. Out of the 54 potential participants initially identified, 29 were excluded due to incomplete documentation or ambiguous imaging. A total of 25 patients were enrolled in the final study cohort.

This retrospective study involved a chart review of pediatric patients with bezoars treated at Chang Gung Memorial Hospital (Linkou branch) from January 2000 to June 2023. Potential participants were identified through a comprehensive database search that included both imaging results (EGD, upper gastrointestinal [UGI] or lower gastrointestinal [LGI] series, and abdominal computed tomography [CT]) and surgical procedural codes related to bezoars. Cases with incomplete documentation or ambiguous imaging that precluded a definitive diagnosis were subsequently excluded (Figure 1). This study was reviewed and approved by the Ethics Committee at Chang Gung Memorial Hospital (IRB No. 2312220018; Date of Approval: 15 April 2024). Demographic characteristics, symptom presentation, imaging findings, and treatment history were extracted from the patients’ medical records.

### 2.2. Data Collection

Data collected from the medical records included demographic information; the duration of symptoms (DOS) before hospitalization; laboratory values, including white blood cell (WBC) counts, sodium (Na) levels, potassium (K) levels, and serum C-reactive protein (CRP) levels; imaging findings; treatment modalities; timing of the operation; the operative results; and complications such as adhesion ileus, residual abscess, and wound complications.

The treatment modalities consisted of surgery, endoscopy, and conservative management. The conservative approach included hydration and the administration of prokinetic or chemical dissolution agents.

Underlying medical conditions were categorized into two primary domains: GI factors and neuropsychiatric factors. “GI factors” were defined as having a history of GI surgery, GI motility disorders, or any congenital or acquired structural abnormalities and diseases of the GI tract. “Neuropsychiatric factors” included established psychiatric or neurodevelopmental diagnoses, specifically autism spectrum disorder, trichotillomania, trichophagia, intellectual disability, and a chronically bedridden status. For patients presenting with multiple concurrent factors, each condition was recorded and analyzed independently within its respective category to ensure a comprehensive assessment of all coexisting comorbidities.

Missing data, including symptom duration, bezoar size, and laboratory values, were handled using a complete-case analysis approach, wherein percentages and statistical comparisons were calculated based on the available valid data for each specific variable.

### 2.3. Outcome Measures

#### 2.3.1. Primary Outcome

The primary outcome was to compare the clinical presentations, laboratory findings, and factors associated with bezoar formation in the GI tract across different bezoar types.

#### 2.3.2. Secondary Outcome

Secondary outcomes included factors related to the requirement of surgical intervention for childhood GI bezoars.

### 2.4. Statistical Analyses

The normality of continuous variables was assessed using the Shapiro–Wilk test. Although a few variables exhibited a normal distribution, the majority of the key clinical parameters deviated from normality. Therefore, to maintain statistical consistency and account for the limited overall sample size (*n* = 25), all continuous variables are uniformly presented as medians with interquartile ranges (IQRs). Inter-group comparisons for continuous variables were performed using the Mann–Whitney *U* test, while categorical variables were analyzed using Fisher’s exact test. A *p* < 0.05 was defined as the threshold for statistical significance. All statistical analyses were conducted using IBM Statistical Package for the Social Sciences (SPSS) version 24.0 (IBM Corp., Armonk, NY, USA).

## 3. Results

### 3.1. Demographic and Clinical Characteristics

A total of 25 patients were enrolled in the study, comprising 14 females (56%) and 11 males (44%). The median age of the patients was 12.0 years (IQR: 5.9–15.0 years). The demographic and clinical characteristics of the patients are summarized in Supplementary Table S1. The number of patients who underwent imaging examinations was as follows: 18 cases for abdominal radiography, 11 cases for abdominal sonography, 6 cases for UGI or LGI series, and 14 cases for abdominal CT. The number of positive findings for each imaging method was as follows: two cases (11.1%) for abdominal radiography, four cases (36.4%) for abdominal sonography, five cases (83.3%) for UGI or LGI series, and fourteen cases (100%) for abdominal CT. Seventeen cases were phytobezoars (68%), five were trichobezoars (20%), and three were lactobezoars (12%). Bezoars were identified in the esophagus in four patients (16%), in the stomach or duodenum in twelve patients (48%), and in the small bowel or colon in nine patients (36%).

**Figure 2:**
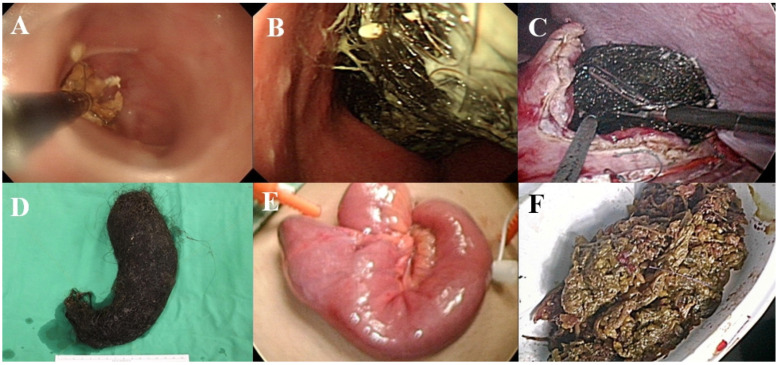
(**A**) Endoscopic view of the phytobezoar with a firm, stone-like texture causing esophageal obstruction. (**B**) Endoscopic view of the trichobezoar in the stomach extending to the duodenum. (**C**,**D**) A gastrotomy was performed (**C**), and the trichobezoar was extracted from the stomach (**D**). (**E**,**F**) An enterotomy was performed (**E**), and the phytobezoar was extracted from the small intestine (**F**).

All four patients with esophageal bezoars had phytobezoars (Figure 2A). The mass shown in Figure 2A was identified as a true bezoar rather than temporary food residue, based on its endoscopic presentation and physical properties. When manipulated and grasped with endoscopic forceps, the specimen exhibited a distinctly firm, stone-like texture and remained completely intact without crumbling or dissolving. This high degree of compaction clearly distinguished it from typical loose food residue. Underlying medical conditions included gastroesophageal reflux disease (GERD) status post fundoplication (one patient), esophageal atresia with tracheoesophageal fistula status post operation (one patient), and alkaline corrosive esophagitis with esophageal stricture (two patients). All patients underwent treatment with EGD.

**Figure 3:**
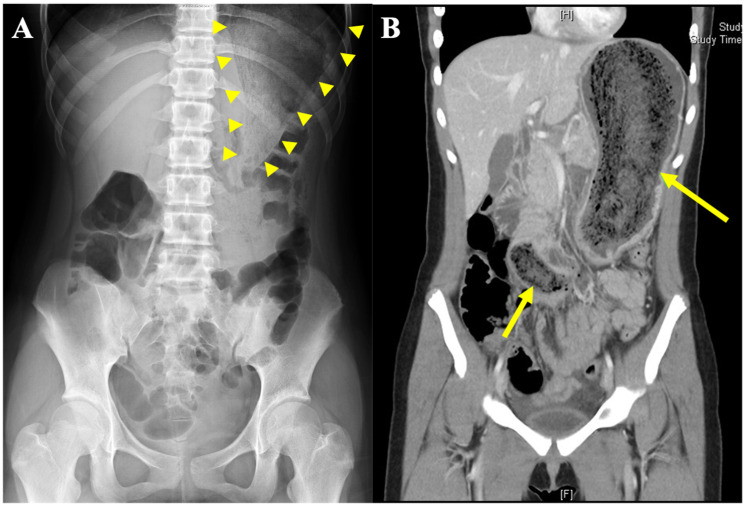
(**A**) Abdominal X-ray of a patient with a trichobezoar, with yellow arrowheads indicating the trichobezoar within the stomach. (**B**) Abdominal CT scan of the same patient, with yellow arrows pointing to the trichobezoar extending through the stomach and duodenum, interspersed with air and mottled calcifications (H: head end; F: foot end).

For patients with bezoars in the stomach and duodenum, the majority had phytobezoars (seven patients, 58.3%) and trichobezoars (four patients, 33.3%) (Figure 2B–D). Figure 3 shows the abdominal X-ray and CT scan of a patient with a trichobezoar. Underlying medical conditions for trichobezoar included trichotillomania and trichophagia (four patients). Underlying medical conditions for phytobezoar included GI factors such as gastric volvulus (one patient), gastric ulcer with deformity (one patient), GERD status post fundoplication (one patient), and superior mesenteric artery (SMA) syndrome (one patient). Neuropsychiatric factors included intellectual disability (two patients), autism (one patient), a history of brain infarction (one patient), and bedridden status (one patient). Half of the patients with bezoars in the stomach and duodenum underwent surgery, with trichobezoar cases being the most prevalent (all four patients underwent surgery).

For patients with bezoars in the small bowel and colon, the majority had phytobezoars (Figure 2E,F). Underlying medical conditions included Meckel’s diverticulum (two patients), a history of ischemic bowel status post operation (one patient), and bowel stenosis (two patients). Almost all patients (88.9%) underwent surgery for treatment.

### 3.2. Differences Between Various Bezoar Groups

Table 1 compares the differences in demographic data, clinical characteristics, laboratory data, and treatment modalities between various bezoar groups. Due to the limited number of cases with lactobezoar, we focused our comparison on the phytobezoar and trichobezoar groups. In terms of gender, all cases in the trichobezoar group were females, while females comprised 41% in the phytobezoar group (*p* = 0.040). We categorized the duration of symptoms into acute and insidious, with one month as the threshold. In the phytobezoar group, data were missing for two patients, accounting for 9% of the total cohort. The proportion of insidious cases in the trichobezoar group was 60%, whereas all cases in the phytobezoar group exhibited an acute presentation (*p* = 0.009).

As for underlying medical conditions, there was a significant difference in GI-related factors, with the phytobezoar group at 76%, including esophageal atresia or stenosis, gastric structural anomalies, or Meckel’s diverticulum, while the trichobezoar group was at 0% (*p* = 0.005). Regarding neuropsychiatric underlying medical conditions, a significant difference was observed, with the trichobezoar group at 100%, as all these patients exhibited trichotillomania and trichophagia. The phytobezoar group, on the other hand, was at 29%, including cases with conditions such as bedridden status, intellectual disability, and autism (*p* = 0.010).

With respect to laboratory values, there were no significant differences in the incidence of leukocytosis (47% vs. 40%, *p* = 1.000) (two missing values in the phytobezoar group, representing 9% of the total cohort), elevated CRP levels (36% vs. 50%, *p* = 1.000) (six missing values in the phytobezoar group and three in the trichobezoar group, collectively representing 41% of the total cohort), and hypokalemia (0% vs. 0%) (two missing values in the phytobezoar group and one in the trichobezoar group, collectively representing 14% of the total cohort) between the phytobezoar and trichobezoar groups. However, the trichobezoar group exhibited a significantly higher incidence of hyponatremia compared to the phytobezoar group (50% vs. 0%, *p* = 0.035) (two missing values in the phytobezoar group and one in the trichobezoar group, collectively representing 14% of the total cohort).

Concerning treatment, the need for surgery was also higher in the trichobezoar cases, at 100%, while 59% of patients with phytobezoar underwent non-surgical interventions (*p* = 0.040). No significant differences were noted in terms of outcomes.

### 3.3. Factors Associated with Surgical Intervention

Table 2 compares the differences between the groups undergoing surgery and those receiving endoscopic or conservative treatment. In terms of location, the group requiring surgery had a higher proportion below the ligament of Treitz, at 57%, while the non-surgical group had a higher proportion above the ligament of Treitz, at 91% (OR = 13.33, 95% CI: 1.32–134.62, *p* = 0.033). Regarding size, the median bezoar size was significantly larger in the surgical group compared to the non-surgical group (13.0 cm [IQR: 8.8–21.4] vs. 3.5 cm [IQR: 2.4–7.9], *p* = 0.005) (one missing value in the surgical group and five in the non-surgical group, collectively representing 24% of the total cohort).

Regarding laboratory values, patients in the surgical group exhibited a higher incidence of leukocytosis (57%) compared to those in the non-surgical group (13%) (three missing values in the non-surgical group, representing 12% of the total cohort). Although this difference did not reach statistical significance (*p* = 0.074), a notable clinical trend was observed (OR = 9.33, 95% CI: 0.89–97.62), suggesting a potential association that warrants further investigation. However, there were no significant differences in the incidence of elevated CRP levels (30% vs. 40%, *p* = 1.000) (four missing values in the surgical group and six in the non-surgical group, collectively representing 40% of the total cohort), hyponatremia (23% vs. 0%, *p* = 0.257), and hypokalemia (0% vs. 0%) between the surgical and non-surgical groups (one missing value in the surgical group and three in the non-surgical group, collectively representing 16% of the total cohort).

## 4. Discussion

Our study revealed several significant findings regarding pediatric bezoars. Phytobezoar emerged as the most prevalent type. Bezoars were found to be distributed throughout the entire GI tract, with the stomach as the predominant location. Furthermore, most patients presenting with bezoars had underlying medical conditions, including GI anomalies and neuropsychiatric disorders. Importantly, our analysis demonstrated that trichobezoars, bezoars located in the lower GI tract, and those of larger sizes were significantly associated with a higher likelihood of surgical intervention.

In this retrospective study, phytobezoars constituted 68% of cases, followed by trichobezoars at 20%, aligning with findings from previous research. In a retrospective study involving 16 pediatric patients, phytobezoars were observed in ten patients (62.5%), with trichobezoar in two patients (12.5%) and lactobezoar in one patient (6.3%) [6]. This concurrence shows the consistency of our results with the existing literature.

Bezoars can be distributed across the entire GI tract. In our study, the preeminent site was the stomach, constituting 44% of cases. Consistent with findings in adults, bezoar formation was observed throughout the GI system, with a notable predilection for the stomach (93.5%) [9]. Similarly, in a pediatric study, the stomach emerged as the predominant site for bezoar identification, accounting for 56% of cases [6]. These collective observations emphasize the stomach’s recurrent significance as a primary locus for bezoar formation across diverse age groups.

Our study found that most of the patients (88%) with bezoars had underlying medical conditions, encompassing GI anomalies and neuropsychiatric disorders. GI anomalies were the predominant contributors to bezoar development, accounting for 60% of cases, suggesting a strong clinical association with bezoar formation, despite the sample size limitations. In a retrospective study focusing on the etiological aspects of bezoar formation, six patients (37.5%) exhibited congenital anomalies in the GI system, including duodenal web, annular pancreas, and Meckel’s diverticulum. Among the neuropsychiatric disorders observed were intellectual disability and trichotillomania [6]. Additionally, in a separate study involving pediatric patients with gastric bezoars, the findings suggested that dysautonomia and underlying GI disorders might serve as potential associated factors [10].

In our study, we observed that individuals with trichobezoars exhibited a distinct female predominance, at 100%. Notably, all these patients manifested trichotillomania and trichophagia, conditions that are more prevalent among females [13]. This observation aligns with a study conducted by HanBin et al., where all 11 patients diagnosed with trichobezoars were female [14]. Similarly, in a study by Habib et al., five out of six patients with trichobezoars were female [15]. These consistent gender associations underscore the correlation between trichobezoar occurrence and female individuals, particularly those with trichotillomania and trichophagia.

In patients presenting with esophageal bezoars, underlying conditions typically include a history of surgery or severe injury resulting in structural or functional abnormalities. Notably, no cases of eosinophilic esophagitis were observed within our cohort. However, previous studies have documented instances of bezoar impaction attributed to eosinophilic esophagitis in pediatric populations [10]. In adults, esophageal bezoars have been reported in patients with esophageal motility disorders, reflux esophagitis, esophageal stenosis, and those with a history of undergoing Nissen fundoplication [16,17,18,19]. Based on our experience, esophageal bezoars can be effectively managed by endoscopic intervention. Therefore, in patients with underlying esophageal diseases presenting with symptoms such as dysphagia or vomiting, early endoscopic examination and treatment are recommended.

For patients with bezoars in the stomach and duodenum, the majority were phytobezoars and trichobezoars. In our study, the majority of patients with bezoars exhibited acute symptoms, whereas a subset of those with trichobezoars presented with insidious symptoms, such as body weight loss or a palpable mass. Trichobezoars, consisting predominantly of fine hair, undergo a relatively slow formation, leading to a more gradual onset of symptoms [20].

However, it is noteworthy that there was no observed evidence of poor growth among our patient cohort. In a retrospective analysis of 11 pediatric patients with trichobezoars, it was found that six individuals presented with a palpable left upper abdominal mass, one case had a weight lower than –2 SD, while four cases fell between –2 SD and –1 SD [14]. Another retrospective study involving six patients with trichobezoars highlighted subtle symptoms, including body weight loss, chronic abdominal distension, and vomiting [15].

There are limited data investigating the clinical significance of leukocytosis and electrolyte imbalances in children with gastrointestinal bezoars. Previous studies have reported that severe leukocytosis is a predictor of strangulated bowel obstruction in pediatric patients [21], and hyponatremia has been observed as a predictor for the need for surgery in children with intussusception [22]. In this study, we compared the leukocytosis and electrolyte values (sodium and potassium) between the phytobezoar and trichobezoar groups and further examined their associations with the need for surgical intervention. Our findings indicated that patients with trichobezoars had a significantly higher rate of hyponatremia (50% vs. 0%, *p* = 0.035, Table 1) compared to those with phytobezoars.

Although the precise reasons for the higher prevalence of hyponatremia in trichobezoar cases remain poorly defined, this trend likely reflects the chronic, insidious nature of the disease. While phytobezoars typically prompt immediate care due to acute obstruction, trichobezoars form over months or years, causing long-standing overlooked fluid loss from recurrent vomiting [12]. Past research has shown that when the body loses a massive amount of intravascular fluid, it will prioritize maintaining blood volume over retaining normal osmolality, which significantly boosts antidiuretic hormone (ADH) secretion [23]. In trichobezoar cases, the prolonged fluid loss likely causes this exact type of severe volume depletion. This triggers a strong release of ADH, causing the body to retain too much water and ultimately leading to dilutional hyponatremia.

Furthermore, our data may suggest a potential association between leukocytosis and the need for surgery. Although the correlation between the requirement for surgical intervention and the development of leukocytosis did not reach statistical significance (*p* = 0.074, exceeding the alpha level of 0.05), a notable clinical trend was observed. Specifically, the substantial effect size (OR = 9.33) suggests a clinically meaningful disparity. This lack of statistical significance is likely due to the limited statistical power constrained by the small sample size. Future larger prospective cohort studies are warranted to clarify the predictive value of white blood cell counts regarding surgical necessity.

In this study, all patients diagnosed with trichobezoars underwent surgical intervention as part of their treatment. Consistent with previous studies involving individuals with trichobezoars, the prevailing approach for most cases has been surgical management [14,15,24]. While a small-sized trichobezoar can be potentially extracted endoscopically using a basket or snare [25,26,27], the removal of gastric trichobezoars often needs surgical intervention. This requirement arises from the tightly interwoven nature of the hairs, which makes them resistant to chemical softening or endoscopic extraction.

Treatment options for phytobezoars currently include attempts at dissolution using cola beverages, papain, or cellulase and extraction using endoscopic devices when the bezoar is located in the upper GI tract. Surgical approaches, such as laparotomy and laparoscopic surgery, are also considered [28,29,30]. In our study, 59% of the patients with phytobezoars showed recovery with non-surgical treatment. Half of them underwent endoscopic treatment, while the remaining patients received conservative treatment.

Phytobezoars and trichobezoars are distinct entities with different etiologies; however, at the time of initial admission, both present as intraluminal masses that require identical diagnostic differentiation and acute management. Therefore, the statistical comparisons here are valuable despite being limited by the small trichobezoar cohort. To ensure validity under these small-sample conditions, we used Mann–Whitney *U* and Fisher’s exact tests, which do not require large-sample assumptions. Consequently, these comparisons are exploratory rather than definitive, serving as a preliminary clinical contrast for pediatric bezoars.

In our series, patients requiring surgical intervention showed a stronger correlation with bezoars located in the lower GI tract. For bezoars located in the upper GI tract, treatment options encompass chemical dissolution and fragmentation, endoscopic removal, and surgical intervention. Nevertheless, in the case of intestinal bezoars causing obstruction or other complications, surgical intervention is the preferred option. Moreover, the size would impact the treatment option. In our cohort, surgical patients were more likely to have larger bezoars, and consistent with previous findings, larger bezoars pose challenges to conservative management [10].

The present study has some limitations that need to be acknowledged. Firstly, as a retrospective study, this design may result in incomplete or erroneous data, which can introduce selection or information bias and limit the generalizability of these specific statistical findings. Secondly, due to the inherent rarity of pediatric bezoars, our study is limited by a relatively small sample size of 25 patients over two decades. Consequently, the statistical analyses, particularly regarding laboratory parameters, may suffer from limited statistical power. We cannot exclude the risk of a type II error, meaning that certain non-significant findings might be attributed to the small cohort size rather than a true lack of clinical association. Therefore, these results should be interpreted as indicative associations rather than definitive predictors.

This limited sample size also explains the extremely wide CIs observed in several of our analyses, which reflect a degree of uncertainty regarding the exact precision of the estimates. While these wide intervals mean the precise effect sizes should be interpreted with caution, the underlying ORs nonetheless show a strong and consistent clinical trend. Therefore, rather than dismissing these findings, they should be interpreted as indicative associations and meaningful preliminary insights that pave the way for larger multi-center studies.

## 5. Conclusions

Phytobezoar is the most frequent type of bezoar in children. Bezoars may be located throughout the entire GI tract, with the most common site being the stomach. Most patients have underlying medical conditions such as GI tract anomalies, a history of GI tract surgery, or neuropsychiatric conditions. Patients with trichobezoars or bezoars located in the lower GI tract, especially those of larger size, are more likely to require surgery for treatment. Ultimately, these findings are intended to serve as a practical guide for clinicians to improve patient management and optimize therapeutic outcomes.

## Figures and Tables

**Table 1:** Differences in demographics, clinical characteristics, and treatment modalities between phytobezoar and trichobezoar.

Variables	Phytobezoar (*n* = 17)	Trichobezoar (*n* = 5)	OR (95% CI)	*p*
Age, years [median (IQR)]	13.6 (6.3–16.3)	12.0 (7.3–13.6)		0.585
Gender [*n* (%)]			-	0.040 *
Male	10 (59)	0 (0)		
Female	7 (41)	5 (100)		
Height (Z-score) [median (IQR)]	−0.58 (−1.38–0.29)	0.19 (−0.32–0.43)		0.032 *
Weight (Z-score) [median (IQR))]	−0.87 (−1.87–0.53)	0.22 (−0.61–0.64)		0.142
BMI (Z-score) [median (IQR)]	−0.29 (−1.57–0.94)	0.135 (−1.16–0.83)		0.893
Symptom duration (*n*) (2 missing values)			-	0.009 *
Acute (<1 month)	15 (100)	2 (40)		
Insidious (>1 month)	0 (0)	3 (60)		
Location [*n* (%)]			0.46 (0.04–5.09)	1.000
Above ligament of Treitz	11 (65)	4 (80)		
Below ligament of Treitz	6 (35)	1 (20)		
Underlying medical conditions				
Gastrointestinal [*n* (%)]	13 (76)	0 (0)	-	0.005 *
Esophageal atresia or stenosis (*n*)	3	0		
Gastric structure anomaly (*n*)	5	0		
SMA syndrome (*n*)	1	0		
Meckel’s diverticulum (*n*)	2	0		
Bowel stenosis (*n*)	1	0		
History of ischemic bowel (*n*)	1	0		
Neuropsychiatric [*n* (%)]	5 (29)	5 (100)	-	0.010 *
Bedridden status (*n*)	1	0		
Intellectual disability (*n*)	2	0		
Autism (*n*)	2	0		
Trichotillomania, trichophagia (*n*)	0	5		
Laboratory values				
Leukocytosis [*n* (%)] (2 missing values)	7 (47)	2 (40)	0.76 (0.10–5.96)	1.000
Elevated CRP [*n* (%)] (9 missing values)	4 (36)	1 (50)	1.75 (0.08–36.29)	1.000
Hyponatremia [*n* (%)] (3 missing values)	0 (0)	2 (50)	-	0.035 *
Hypokalemia [*n* (%)] (3 missing values)	0 (0)	0 (0)	-	-
Treatment			-	0.040 *
Surgery [*n* (%)]	7 (41)	5 (100)		
Laparotomy (*n*)	5	3		
Laparoscopy (*n*)	2	2		
No surgery [*n* (%)]	10 (59)	0 (0)		
Endoscopy (*n*)	5	0		
Conservative Tx (*n*)	5	0		
Outcome				
Length of hospital stay, days [median (IQR)]	6 (4–13)	9 (8–12)		0.237
Morbidity [*n* (%)]	2 (12) (wound infection)	0 (0)	-	1.000
Mortality [*n* (%)]	0 (0)	0 (0)	-	-

* Numerical and categorical data were analyzed using the Mann–Whitney *U* test and Fisher’s exact test, respectively; a *p* value < 0.05 was considered statistically significant.

**Table 2:** Clinical features related to the need for surgical intervention.

Variables	Surgery (*n* = 14)	No Surgery (*n* = 11)	OR (95% CI)	*p*
Location [*n* (%)]			13.33 (1.32–134.62)	0.033 *
Above ligament of Treitz	6 (43)	10 (91)		
Below ligament of Treitz	8 (57)	1 (9)		
Size [median (IQR)] (6 missing values)	13.0 (8.8–21.4)	3.5 (2.4–7.9)		0.005 *
Underlying medical conditions				
Gastrointestinal [*n* (%)]	7 (50)	8 (73)	0.38 (0.07–2.03)	0.414
Esophageal atresia or stenosis (*n*)	0	3		
Gastric structure anomaly (*n*)	1	4		
SMA syndrome (*n*)	1	0		
Meckel’s diverticulum (*n*)	2	0		
Bowel stenosis (*n*)	2	0		
History of ischemic bowel (*n*)	0	1		
Congenital short bowel (*n*)	1	0		
Neuropsychiatric [*n* (%)]	8 (57)	3 (27)	3.56 (0.65–19.41)	0.227
Bedridden status (*n*)	1	1		
Intellectual disability (*n*)	1	1		
Autism (*n*)	1	1		
Trichotillomania, trichophagia (*n*)	5	0		
Laboratory values				
Leukocytosis [*n* (%)] (3 missing values)	8 (57)	1 (13)	9.33 (0.89–97.62)	0.074
Elevated CRP [*n* (%)] (10 missing values)	3 (30)	2 (40)	0.64 (0.07–6.06)	1.000
Hyponatremia [*n* (%)] (4 missing values)	3 (23)	0 (0)	-	0.257
Hypokalemia [*n* (%)] (4 missing values)	0 (0)	0 (0)	-	-

* Numerical and categorical data were analyzed using the Mann–Whitney *U* test and Fisher’s exact test, respectively; a *p* value < 0.05 was considered statistically significant.

## Data Availability

Due to ethical and confidentiality constraints, the data supporting the findings of this study are restricted. Interested researchers may contact the corresponding author to request access.

## Supplementary Materials

The following supporting information can be downloaded at: https://www.mdpi.com/article/10.3390/children13070929/s1, Table S1: Demographic data, clinical presentations, location, size, underlying medical conditions, and treatment modalities of various bezoars in children.

## Abbreviations

The following abbreviations are used in this manuscript:

GI	Gastrointestinal
EGD	Esophagogastroduodenoscopy
UGI	Upper gastrointestinal
LGI	Lower gastrointestinal
CT	Computed tomography
DOS	Duration of symptoms
WBC	White blood cell
Na	Sodium
K	Potassium
CRP	C-reactive protein
IQR	Interquartile range
CI	Confidence interval
GERD	Gastroesophageal reflux disease
P	Phytobezoar
T	Trichobezoar
L	Lactobezoar
Vomit.	Vomiting
Abd.	Abdominal
BW	Body weight
S/S	Symptoms/signs
S/p	Status post
SMA	Superior mesenteric artery
OR	Odds ratio
ADH	Antidiuretic hormone
