# Supplementary material for: Gastrointestinal Bezoars in Children: Clinical Characteristics, Contributing Factors, and Treatment Outcomes
Source: Children (Basel). 2026 Jul 15;13(7):929. doi: 10.3390/children13070929 (PMC13406977; doi:10.3390/children13070929)
Supplement: Supplementary file 1 [file children-13-00929-s001.zip › children-4379527-supplementary.pdf]

Table S1. Demographic data, clinical presentations, location, size, underlying medical conditions, and treatment modalities of various bezoars in children

Gender	Age(yrs)	Type	Symptoms	S/S duration	Location	Size(cm)	Underlying medical conditions	Treatment
Male	6.8	P	Vomit.	1 day	Esophagus	-	GERD s/p fundoplication	EGD
Male	2.3	P	Vomit.	5 days	Esophagus	1	Esophageal atresia and TE fistula s/p operation	EGD
Male	5.5	P	Dysphagia	1 day	Esophagus	2.9	Alkaline corrosive esophagitis, esophageal stricture	EGD
Female	16.4	P	Dysphagia	1 week	Esophagus	-	Alkaline corrosive esophagitis, esophageal stricture	EGD
Female	14.8	P	Vomit., abd. pain	3 days	Stomach	9*6*3.7	-	EGD
Male	7.1	P	Vomit., abd. distension	1 week	Stomach	-	Chromosome abnormality, intellectual disability, gastric volvulus	Surgery
Female	12.8	P	Abd. pain	-	Stomach	-	Gastric ulcer with deformity	Conservative
Female	14.9	P	Vomit.	-	Stomach	4	GER s/p fundoplication, IBS, infantile autism, ADHD	Conservative
Female	13.6	P	Abd. pain, poor appetite	5 days	Stomach	-	Seizure disorder, intellectual disability, gastritis	Conservative
Male	10.7	L	Vomit., poor feeding	-	Stomach	-	Moyamoya disease with brain infarction, respiratory failure s/p tracheostomy	Conservative
Female	12.1	T	Vomit., Abd. pain	2 days	Stomach, duodenum	22.7*6.5*5.2	Trichotillomania, trichophagia	Surgery
Female	12.0	T	Vomit., poor appetite, abd. pain, BW loss	2 months	Stomach, duodenum	20*5*4	Trichotillomania, trichophagia	Surgery
Female	15.0	T	Palpable abd. mass	2 years	Stomach, duodenum	13*12.5	Trichotillomania, trichophagia	Surgery
Female	5.4	T	Vomit., abd. pain, epigastric nodule	1 month	Stomach, duodenum	10*4*4	Trichotillomania, trichophagia	Surgery
Female	17.7	P	Vomit., abd. distension, poor appetite	2 days	Stomach, duodenum	Stomach part: 12<br>Duodenum part: 4	Rett's syndrome with bedridden, SMA syndrome	Surgery
Female	1.2	P	Vomit.	4 days	Duodenum	3	-	Conservative
Female	9.2	T	Vomit., abd. pain, poor appetite	3 days	Small bowel	7.5*2.7*2.0	Trichotillomania, trichophagia	Surgery

Male	16.3	P	Vomit., abd. pain	1 day	Small bowel	15*3.8	Meckel's diverticulum	Surgery
Male	14.6	P	Abd. pain	1 day	Small bowel	34.3*3.9	Epilepsy, autism	Surgery
Male	6.4	P	Abd. pain, abd. distension	1 day	Small bowel	2*0.9*1.8	-	Surgery
Male	18.0	P	Vomit., abd. pain	2 days	Small bowel	30*3.5	Meckel's diverticulum	Surgery
Male	18.0	P	Abd. pain	2 days	Small bowel	7.5*2.6	Internal herniation with ischemic change s/p operation	Conservative
Female	0.1	L	Vomit.	3 weeks	Small bowel	6.1*2.3	Ileal stricture	Surgery
Female	0.5	L	Vomit., poor appetite	3 days	Small bowel	15*4	Congenital short bowel	Surgery
Male	6.3	P	Vomit., abd. pain	2 days	Small bowel, colon	12*6	Colon stenosis	Surgery

Abbreviations: P: phytobezoar; T: trichobezoar; L: lactobezoar; EGD: esophagogastroduodenoscopy; Vomit.: vomiting; Abd.: abdominal; BW: body weight; S/S: symptoms/signs; s/p: status post.
